# Miscellany

**Published:** 1903-06

**Authors:** 


### Miscellany.

Crystallized Peroxide of Hydrogen.[1]—The firm of E. Merck has been supplying a solution of peroxide of hydrogen at thirty per cent. for some years. This peroxide fulfils all requirements as regards purity and stability. This preparation is appreciated as an antiseptic, more especially in connection with surgical instruments and appliances.

Peroxide of hydrogen, contrary to recent assertions, crystallizes with care in a very distinct manner. The preparation used contained from ninety-five to ninety-six per cent. of hydrogen dioxide. In a freezing mixture at —20° it remained liquid; of ether and solid anhydride it solidified into a hard resisting mass.

The refrigeration must be sufficient to the evolution of heat which takes place. If the operation is not conducted under these conditions oxygen very rich in ozone is given off.

The most interesting reaction takes place with sulphate of

---

[1] From Zeitschrift für angewandte Chemie. By William Staedel.

titanium. By its help we are able to detect 1 part of peroxide of hydrogen in solution in 1,800,000 parts of water. When the ratio is 1 to 18,000 the reaction is manifested by the appearance of a deep yellow coloration, becoming bright yellow for 1 in 180,000.

Peroxide of hydrogen is capable of forming crystallizable compounds with metallic salts. The compound containing cadmium lends itself very well to the demonstration. On pouring a ninety to ninety-five per cent. solution of peroxide of hydrogen into a concentrated solution of chloride of cadmium the mass becomes thickened by the formation of small, fine, silky flakes. When collected and dried they contain about twenty-three per cent. of the peroxide of hydrogen.

From the point of facts already known we may say with safety that the use in surgery of an antiseptic product incapable of introducing foreign matters into wounds, or of producing anything besides water and oxygen by its decomposition, is made practicable by the present preparation. Anhydrous peroxide of hydrogen appears to be easily transported. A well-packed case was placed on a truck and sent on a journey of fifty or sixty kilometres without any change.

---

A Suggestion.—A filling in a tooth is only the restoration by mechanical means of the integrity of the enamel coating in form and continuity. That is, an indestructible something is put into a defect in this enamel coating so accurately that if the natural movements of teeth, jaws, tongue, lips, and saliva cannot keep it clean we may supplement nature by our brush and floss and washings, so that the teeth *may* be kept clean. For a clean tooth never decays, and a clean tooth has no tartar upon it; so pyorrhœa alveolaris is not known in the presence of entire cleanliness.—E. H. Bogue.

---

A Method for backing Porcelain Teeth for Metal Work.—Hold a piece of 30-gauge platina plate flat against the tooth with an edge touching the side of a pin, mark a line from the pin across the metal, then place the platina flat against the tooth under the pins and mark two lines from the pins across the first line. Punch holes where the lines intersect; place backing and file flush with the edge of the tooth all around; then remove platina and adapt "crown metal" (pure gold and platina rolled together) two-one-thousandths of an inch, by first marking with the pins an impression in the foil, then puncture holes with a sharp-pointed instrument, then press to place upon the tooth, and with an instrument having a hole to telescope over the pins, with a turn burnish the extended edges of the punctured holes around pins, thus making a water-tight joint. Have the foil—gold surface always next the porcelain—extend beyond the edge of the tooth the sixteenth of an inch and cover the end fitting the cap (if a crown); then replace the platina backing and hold by splitting the previously cut pins.

The free edge of the foil should be drawn towards the backing at a slight angle, or at a right angle to the backing. The foil extending over the end of the facing next the cap protects it from borax, draws the solder, and reflects a better shade at the joint in front. The free edge of foil at the mesial, distal, and incisal edges protects the tooth from the flame and borax, where, with the old form of backing it is often exposed in trimming the investment. The matrix-like box thus formed with the cap draws the solder to such shape as to allow in finishing with the least labor the imitation of the outline of the palatal surface of incisor teeth, a detail never observed.—P. B. McCullough.

---

Salol as a Mouth-Wash.—James has made a series of experiments with a proprietary emulsion of salol, known as odol, to determine whether it is broken up into its active constituents in the mouth. He has shown that it is decomposed by the saliva, by the mucous membrane, and by various bacteria, thus slowly liberating carbolic and salicylic acids.—H. C. W., *American Medicine.*

---

Neurasthenic Neuralgias.—E. Jendrassik calls attention to the need for greater care in the diagnoses of neuralgias, particularly in the differentiation of those cases of pseudo-neuralgias or neurasthenic neuralgias frequently attributed to some non-existent cause of irritation, a supposed bone splinter or imperfectly filled tooth, forms of neuralgia referred to by Paul Blocq under the name topoalgias, or by Galippe as dental obsessions. These pseudo-neuralgias, described by Blocq as monosymptomatic neurasthenias, are to be recognized not only by the neurasthenic symptoms and heredity, but by the absence of the well-known symptoms of true neuralgia, such as periodicity, flushing of the face, involuntary shedding of tears, etc., all of which fail in the neurasthenic variety.—C. S. D., *American Medicine.*

---

Definition for Articulation.—The four positions of the lower jaw in its relation to the upper, from a strictly dental stand-point, are occlusion (being the relation desired in taking the "bite"), right lateral occlusion, left lateral occlusion, and incisal occlusion. The combination of these four positions in action constitutes articulation.—P. B. McCullough.

---

Coloring Artificial Teeth.—In the natural tooth the greater portion is dentine, and that is always yellow and extends nearly to the tip of the tooth. The blue or gray shade is in the enamel, and its intensity depends upon its thickness, sometimes one-eighth of an inch of the end being solid blue. But above this and all through the substance, beneath the enamel, should be the yellow body, light or dark, according to the necessity of the case.

Were this principle carried out in artificial teeth, a natural effect would result, now so lacking in a large proportion of cases.—Dr. L. P. Haskell, *Dental Review.*

---

A New Local Anæsthetic discovered.—Hungarian dentists and chemists claim to have discovered a valuable *local anæsthetic,* an alkaloid nervocidine, the hydrochloride of which is stated to have similar properties to cocaine, but to produce a much more lasting anæsthesia. The base is obtained from the Indian plant, "Gasu Basu," the properties of the leaves of which were first discovered by D. Dalma, who successfully employed them in painful pulpitis, with such good results that he reported that the drug might displace arsenic for dental purposes. B. von Fenyvessy has investigated the properties of the alkaloidal hydrochloride, as prepared by Dalma, which is a yellow amorphous, hygroscopic powder, readily soluble in water. It produces marked anæsthesia of the cornea in 0.1 or 0.2 per cent. solution, which is very persistent, and a 0.1 per cent. solution brushed on the mucous membrane of the cheek also gives marked anæsthesia. Stronger solutions, exceeding 0.5 per cent., produce irritation of the cornea, and a two per cent. solution causes ulcerative keratitis in dogs and rabbits, which lasts ten days, during which period the anæsthesia also lasts. It does not appear to produce anæsthesia by subcutaneous injection. Its general effect is that of a paralyzing poison. Although its anæsthetic effect is much more prolonged than that of cocaine, the length of time before this effect supervenes, the irritation caused by the drug, and the toxic symptoms it produces do not point to the probability of its being of general service, except perhaps in dental practice.—*Lancet.*

---

Polishing Porcelain.—As perfect a polish may be given porcelain as can possibly be given gold or any of the metals. After grinding the porcelain, go over the surface with a sand-paper disk, following with a cuttle-fish disk. Then take oxide of tin polishing putty, and with wooden points, known as Barker's porous polishers, as beautiful a surface may be given to porcelain as can be imparted to it by fusing. By this method one may have the assurance that a perfect polish may be given inlays without fusing again.—Dr. C. F. Hartt, *Dental Review.*

---

Cement Anchorage for Amalgam Fillings.—Remove carious dentine and properly prepare the cavity. Line the cavity with thin cement, being careful not to use an excess. Place a piece of amalgam in the cavity and press to place, forcing it into close contact with the cavity walls. This will crowd out any excess of cement. Pack more amalgam until the margins of the cavity are neared, then clear them of any cement, so that only amalgam will be in contact with them. Then finish the filling as usual, applying a matrix, if necessary. The cement, besides securely anchoring the filling, which is of great value in strengthening a frail tooth, also prevents the dark discoloration so often seen in teeth filled with amalgam.—McClain.

Bleaching an Anterior Tooth.—In a clinic at the Chicago College of Dental Surgery Alumni Association, Dr. J. P. Buckley gave his method of bleaching an anterior tooth with sodium dioxide as follows:

The dam is placed over the tooth to be bleached and the adjacent teeth. A thin platinum band is placed about the tooth to be bleached and white gutta-percha warmed and pressed about it to form a pocket about the cavity. By the use of a small gold or platinum spoon the powder is placed in the cavity, and with a glass instrument is forced down into it and into the upper third of the root. Distilled water is now dropped into the cavity. A platinum ribbon is held over the cavity, forcing the generated oxygen into the dentine. This is followed by removing the treatment, washing and drying the cavity and repeating the operation as may be required to satisfactorily bleach the tooth. Should it be found impossible to remove the pigment mechanically with water, a solution of three per cent. sulphuric acid is used to chemically dissolve it, after which wash with water and let dry without using hot air preferably.

Now burnish a paste made of precipitated calcium phosphate and distilled water into the lower third of the root-canal and against all exposed dentine. Make a base for final filling, using a light-colored cement.—*Dental Review.*

---

Dental Inspection in Schools.—Ensch and Polus call attention to the importance of health boards giving attention to such diseases of children in schools as are readily relieved, as dental caries, adenoid vegetations, affections of the ears and eyes, and refers to the very satisfactory results obtained by the special dental service of Brussels organized in 1877, and the work of various individuals in other European cities. The ten commandments of dental hygiene proposed by Dr. Röse, of Munich, are commended to the attention of hygienists and parents: 1. Forget rather to wash the body than to wash the mouth and clean the teeth. 2. Accustom children to the pleasure to be derived from dental hygiene. That which is neglected in youth is never retrieved later. The care of the milk teeth is no less important than that of the permanent teeth. 3. Avoid sweets and very soft foods. The best means of preventing caries is the vigorous chewing of bread with thick crust. 4. Never forget to clean the teeth at night. He who defers cleansing the mouth till morning resembles one who shuts the stable door after the horse is stolen. 5. Mechanical cleansing with the aid of a brush constitutes the basis of all artificial hygiene. 6. Inoffensive antiseptic mouth-washes and a good tooth-powder are very efficacious for completing the buccal and dental hygiene. 7. Have the teeth examined twice a year by a dentist, who will discover and check the spread of decay. 8. Have the tartar removed from time to time. 9. Old roots which cannot be saved by treatment should be removed, whether painful or not. 10. Mothers should give preference to foods rich in nutritive salts (green vegetables, milk, eggs, etc.) before the birth of their children and during lactation; and the child during earlier years should take the same with a view to greatest possible development of the teeth.—C. S. D., *American Medicine.*

---

A New Antiseptic.—Septoforma is a condensation product of formaldehyde,[1] with the terpen-naphthalin-phenol group, which by means of an alcoholic potassium oleate forms a yellowish noncaustic solution in water. While it softens the skin, it is not soapy, and even the concentrated solution does not redden or irritate. According to Engels, a three per cent. solution destroys staphylococcus pyogenes aureus in three minutes, the cholera vibrio in one minute, and the typhoid bacillus in ten minutes. Instruments of various metals were exposed for three days to thirty per cent. solution of septoforma, and none of them showed the slightest change except those made of aluminum. It was first introduced through veterinary surgery, but has been used by several observers in gynæcologic work and in general surgery. It seems to have advantages over other similar disinfectants, owing to a much less penetrating odor, absence of local irritation or injury to metal instruments, and that it has a considerable degree of germicidal power. It is recommended for the disinfection of instruments in the strength of five per cent. to ten per cent. solution and as a wash for wounds in a three per cent. solution. It is also useful in various skin affections and in suppurative ulcers in the form of a ten per cent. ointment with lanolin, with which its deodorizing property makes it of special value.—H. C. W.

---

[1] Die med. Woch., October 6, 1902, 414.

